# Multiscale Modeling of Agglomerated Ceria Nanoparticles: Interface Stability and Oxygen Vacancy Formation

**DOI:** 10.3389/fchem.2019.00203

**Published:** 2019-05-22

**Authors:** Byung-Hyun Kim, Jolla Kullgren, Matthew J. Wolf, Kersti Hermansson, Peter Broqvist

**Affiliations:** ^1^Department of Chemistry—Ångström Laboratory, Uppsala University, Uppsala, Sweden; ^2^Platform Technology Laboratory, Korea Institute of Energy Research, Daejeon, South Korea

**Keywords:** multiscale modeling, density functional theory, self-consistent charge density functional tight binding, reducible oxides, cerium dioxide, nanoparticles, agglomeration

## Abstract

The interface formation and its effect on redox processes in agglomerated ceria nanoparticles (NPs) have been investigated using a multiscale simulation approach with standard density functional theory (DFT), the self-consistent-charge density functional tight binding (SCC-DFTB) method, and a DFT-parameterized reactive force-field (ReaxFF). In particular, we have modeled Ce_40_O_80_ NP pairs, using SCC-DFTB and DFT, and longer chains and networks formed by Ce_40_O_80_ or Ce_132_O_264_ NPs, using ReaxFF molecular dynamics simulations. We find that the most stable {111}/{111} interface structure is coherent whereas the stable {100}/{100} structures can be either coherent or incoherent. The formation of {111}/{111} interfaces is found to have only a very small effect on the oxygen vacancy formation energy, *E*_vac_. The opposite holds true for {100}/{100} interfaces, which exhibit significantly lower *E*_vac_ values than the bare surfaces, despite the fact that the interface formation eliminates reactive {100} facets. Our results pave the way for an increased understanding of ceria NP agglomeration.

## Introduction

Synthesized ceria samples often appear in the form of agglomerates of nanoparticles (NPs) (Wang and Feng, 2003; Mai et al., 2005; Du et al., 2007; Lin et al., 2012; Liu et al., 2015; Schlick et al., 2016; Lykaki et al., 2017). In most cases, agglomeration degrades the total catalytic performance due to the elimination of reactive surfaces (Li et al., 2003; de Diego et al., 2005). However, in some cases, agglomeration has been found to enhance the catalytic performance due to favorable properties of the interface itself, e.g., by facilitating the formation of critical defects (Maillard et al., 2005). For ceria, only a small fraction of the numerous published studies in recent years have scrutinized the nature of native interfaces. On the experimental side, Wang and Feng (2003) synthesized ceria NPs of truncated octahedral shape and of sizes of 3–10 nm, and noted that the agglomeration of their ceria NPs could be correlated with the disappearance of accessible {100} facets during growth. The resulting agglomerates consisted of NPs in the range of ~1 μm in diameter and with each consecutive NP pair forming a coherent interface, i.e., perfectly matching their lattices across the interface by sharing a common {111} facet. Reactivity was not discussed in that study, but in a series of papers, Hojo et al. (2010) and Feng et al. (2012, 2016) reported that the atomic structure and local oxygen dynamics are modified by the formation of grain boundaries in thin-films of ceria. They also concluded that grain boundaries can be stabilized by the presence of oxygen vacancies.

On the theoretical side, Sayle and co-workers have used force-field simulations to investigate structure-property relationships for ceria nanostructures and have for example investigated nanocubes, nanorods, nanochains, and mesoporous structures (Sayle et al., 2005, 2011; Jeyaranjan et al., 2018). In one of their studies, ceria nanochains were generated via oriented attachment (OA) and used as model systems to explain the mechanism for ceria nanorod formation (Sayle et al., 2011). Sk et al. (2014) performed density functional theory (DFT) calculations for self-assembled one and two dimensional nanoceria networks. Thus, they studied nanowires and nanogrids built from corner-sharing Ce ions or from sharing of oxygen terminated {100} facets; the latter requiring an energy cost to reduce one side of the {100} facets of the NPs prior to agglomeration. For future reference, we note that the periodic boundary conditions used in Sk et al. (2014) induce constraints on the possible NP interfaces that can be modeled. To the best of our knowledge, detailed theoretical investigations at the atomic scale of *aperiodic* ceria NP interfaces and their redox activity are lacking in the scientific literature. The present study primarily deals with the interface between two ceria NPs and the specific oxygen chemistry that may accompany it.

In this work, we will show that stable ceria-ceria interface structures formed between agglomerated ceria NPs display oxygen redox properties that are quite different from those of either the bare surface or the bulk material, and that this is true both for the “rim” region and the “internal” region of the interface, and both for *coherent* and *incoherent* interfaces. Here the term “rim” refers to interface regions where the constituent atoms are exposed while the term “internal” refers to regions where they are not. Our model systems are ceria NP pairs, with an outlook toward chains at the end of the article.

Our method is a *sequential multiscale simulation approach* linking three levels of theory along the multiscale ladder, namely Hubbard augmented DFT (DFT+*U*) (Perdew et al., 1996; Dudarev et al., 1998), the self-consistent-charge density functional tight-binding approach (SCC-DFTB+*U*) (Elstner et al., 1998; Aradi et al., 2007; Kullgren et al., 2017), and finally, for stoichiometric NPs, also the reactive force-field (ReaxFF) method (van Duin et al., 2001; Broqvist et al., 2015). The SCC-DFTB method is an approximate DFT method, which combines an electronic structure description at the minimal basis-set level with a Coulombic expression for long-range interactions. The charges are of Mulliken type and are calculated self-consistently (SCC) in the simulation depending on the elements and the geometric structure. The ReaxFF method, finally, is an advanced bond-order-dependent force-field method, which allows for the description of dissociation and formation of chemical bonds in various kind of compounds.

Moving from DFT to SCC-DFTB to ReaxFF implies a scheme taking us from fine-grained toward more coarse-grained materials models, namely from electrons to atoms in the prevailing MODA language's model entities (Anne de Baas, 2018), as promoted within the European Materials Modeling Council (EMMC, 2019). We use a consistent parameterization of the SCC-DFTB and ReaxFF interaction models which are both trained against DFT+*U* data. These parameterization efforts were described in detail in previous publications (Broqvist et al., 2015; Kullgren et al., 2017). In the present paper, however, we mostly move in the opposite direction, from SCC-DFTB to DFT, using the former method to screen many NP assembly structures, the most stable of which are subsequently scrutinized using DFT. Finally we make use of the ReaxFF model for ceria to perform long MD simulations during the initial stages of nanoceria agglomeration. In the following, we will often omit the *U* term in our notation for DFT and SCC-DFTB not to burden the text, but it is in fact present in all our electronic calculations of (partially) reduced ceria systems.

We will present the stable ceria interface structures found and their effects on the oxygen vacancy formation energy, which we will use as a key descriptor of oxygen chemistry. We find that while agglomeration of course lowers the NPs' surface areas, the interface-specific defects might lead to an activation of agglomerated NPs toward redox processes. Finally, we also discuss how to extend our findings into larger interface systems using the ReaxFF method. Here we will briefly address the mechanism of NP agglomeration and relate to the concept of OA which has been amply discussed in the literature (Si et al., 2006; Du et al., 2007; Lin et al., 2012; Raju et al., 2014; Zhang and Banfield, 2014), and where nano-crystallites collide and interact favorably, e.g., by sharing some common crystallographic orientations, resulting in the formation of interfaces between the NPs.

## Interface Construction Protocol and Computational Methods

In this work, as mentioned, we use a computational protocol based on a combination of electronic structure methods, namely the SCC-DFTB and DFT methods, to study ceria NP interface structures. SCC-DFTB is roughly 2 orders of magnitude faster than standard (semilocal, i.e., “GGA”-type) DFT calculations. With a proper parameterization, the accuracy with respect to the training set will be close to that of the underlying DFT method. This agreement will be reported in the section Consistency Between the Methods That We Have Used. The considerable speedup compared to DFT makes SCC-DFTB a valuable tool for the investigaton of a large number of interface structures, which is exactly the way we have used it here.

The SCC-DFTB calculations were performed using the DFTB+ code (Elstner et al., 1998; Aradi et al., 2007). The parameters describing the Slater-Koster tables and repulsive potentials for ceria have been generated and thoroughly tested previously (Kullgren et al., 2017). DFT calculations in the implementation with plane waves and pseudopotentials were performed using the Vienna *ab-initio* simulation package (VASP) (Kresse and Hafner, 1993, 1994; Kresse and Furthmüller, 1996a,b). The exchange correlation energy was described within the generalized gradient approximation (GGA) following the work of Perdew-Burke-Ernzerhof (PBE) (Perdew et al., 1996). Pseudopotentials of the Projector Augmented Wave (PAW) type were used to describe the core electrons (Blöchl, 1994; Kresse and Joubert, 1999). The Kohn-Sham single-electron wave functions were expanded by plane waves with a cut-off energy of 408 eV. To accurately treat the strongly correlated Ce 4*f* states, the rotationally invariant Hubbard correction scheme from Dudarev et al. (1998) with a *U*-value of 5 eV on the Ce *4f* states was applied. Previous studies have shown that this *U*-value gives a good description of the electronic properties of reduced ceria (Castleton et al., 2007; Loschen et al., 2008; Kullgren et al., 2013; Du et al., 2018). In all calculations, the Brillouin zone was sampled at the Γ-point. All structures were pre-optimized using SCC-DFTB. Some were selected and subsequently geometry optimized at the DFT level of theory. Geometry optimizations for all modeled structures were carried out until the maximal force acting on any one atom was <0.02 eV/Å.

**Figure 1:**
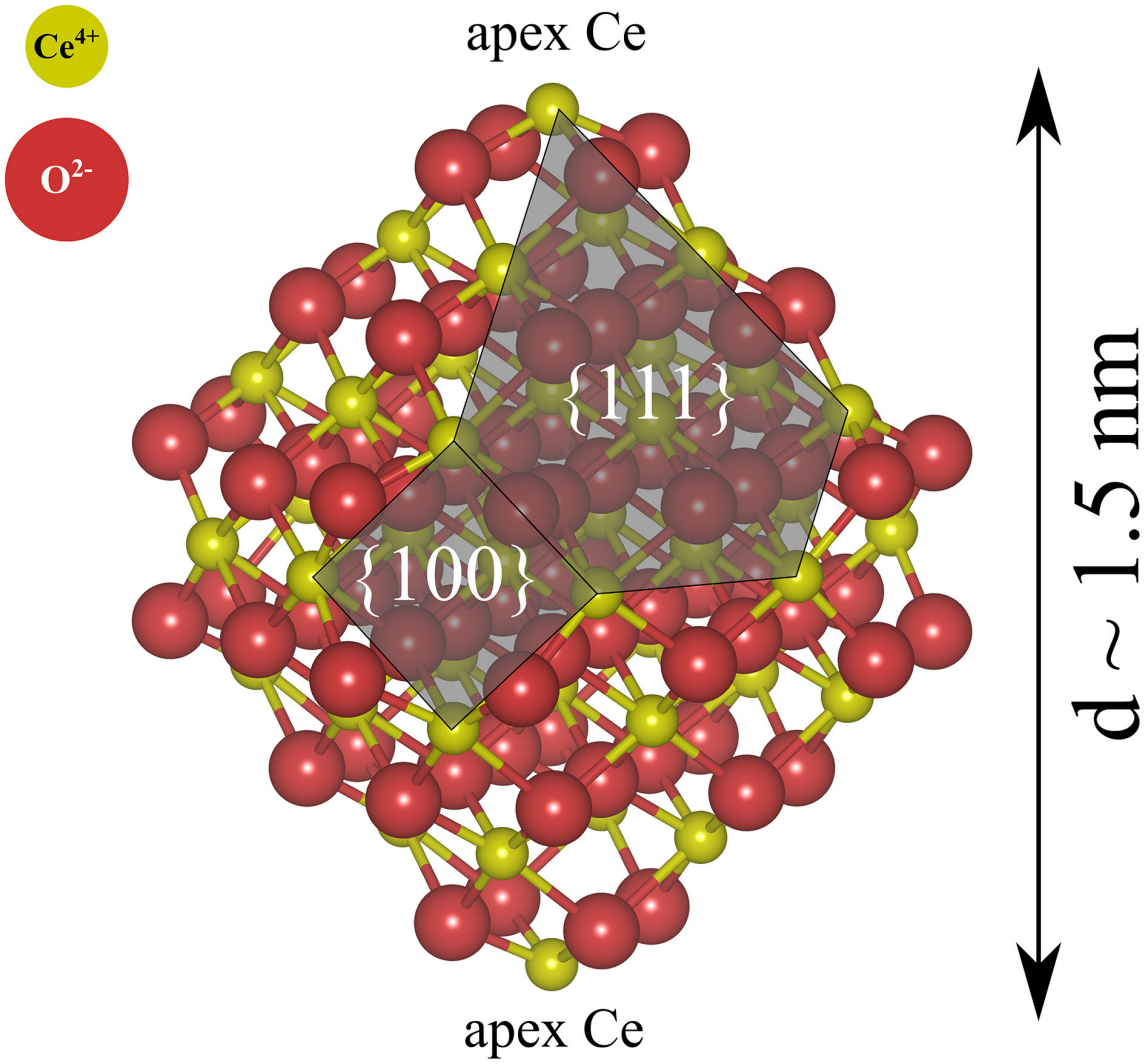
Atomic structure of a stoichiometric truncated octahedral Ce_40_O_80_ NP optimized at the DFT+*U* (*U* = 5 eV) level in this work. Red and yellow spheres indicate O^2−^ and Ce^4+^, respectively.

We have primarily studied NP pairs, (NP)_2_, formed by two stoichiometric truncated octahedral ceria NPs with the chemical formula Ce_40_O_80_. The two NPs were connected via low-index facets, forming {111}/{111} or {100}/{100} interfaces. The *free* solid-vacuum {111} surfaces of stoichiometric ceria are known to be the most stable ones and they make up the largest surface area on the isolated Ce_40_O_80_ particles (see Figure 1). *Free* {100} surfaces are the most reactive low-index surfaces of ceria and have been found to be present on truncated octahedral NPs in UHV (Zhang et al., 2011; Lin et al., 2014).

To generate representative NP interfaces we placed two identical NPs, facet to facet at a distance of 2.7 Å in a periodic cell of the dimensions, 4.5 × 3 × 3 nm^3^. Reference calculations were performed for the isolated Ce_40_O_80_ NP, placed in a 3 × 3 × 3 nm^3^ box. One such particle is approximately 15 Å in diameter. We used rather large cell sizes to prevent image-image interactions between particles in neighboring supercells. The same supercell sizes were used in both the DFT and the SCC-DFTB calculations. The final optimized structures were analyzed in terms of stability, structural parameters and electronic properties, as defined in the Results and Discussion section. The calculations for the O_2_ molecule in its ground triplet electronic state were also performed, using a large periodic cell, 3 × 3 × 3 nm^3^.

In summary, the following stoichiometric systems are examined by electronic structure methods in this study: an isolated truncated Ce_40_O_80_ labeled NP (Figure 1), and a number of (Ce_40_O_80_)_2_ systems labeled (NP)_2_. Reduced systems, where one neutral O atom was removed from (various locations on) the ceria particle to form half an oxygen molecule, were also studied for both the NP and the (NP)_2_ systems.

## Results and Discussion

### Consistency Between the Methods That We Have Used

**Figure 2:**
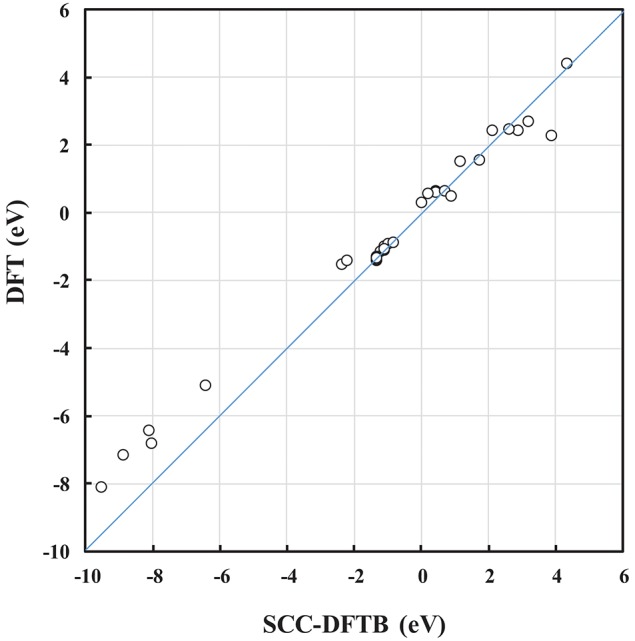
Correlation plot between SCC-DFTB and DFT calculated energies, such as the *E*_interface_ and *E*_vac_ of NP and (NP)_2_ systems.

The DFTB parameters (the entries in the Slater-Koster table and the V_rep_ parameters) used here were trained against DFT calculations (Kullgren et al., 2017). In Kullgren et al. (2017), the DFTB model was subjected to an extended validation process, and passed the test of reproducing the reference DFT results with respect to NP structures as well as oxygen vacancy formation energies in ceria bulk and at the low-index surfaces. In the present paper, the DFTB model was further validated for some of the interface structures, and we find that calculated energies, such as the interface formation energy, *E*_interface_, and the oxygen vacancy formation energy, *E*_vac_, from SCC-DFTB are in reasonably good agreement with those from DFT (see the correlation in Figure 2 and Table S-I). Moreover, the optimized DFT and SCC-DFTB structures are found to differ by <1% in terms of bond lengths. This consistency is a great advantage in our approach to generating interface models, as it reduces the number of computationally expensive DFT energy evaluations. We find that the computational approach used here, i.e., pre-screening using SCC-DFTB followed by full structural optimisations using DFT for selected systems is an efficient and useful protocol.

### Identifying Stable Coherent and Incoherent Interfaces

For the single NP, we use a prototypical model structure (Figure 1), namely a stoichiometric Ce_40_O_80_ particle, which is a truncated octahedron exposing eight large {111} facets and six small {100} facets, two of which are decorated with an apex Ce ion. Ceria NPs exhibiting truncated octahedral shapes have been reported in many experiments (Wang and Feng, 2003; Mai et al., 2005; Du et al., 2007; Tan et al., 2011; Wang and Mutinda, 2011; Lin et al., 2012; Cordeiro et al., 2013; Florea et al., 2013; Liu et al., 2015) and previous theoretical studies have shown the truncated octahedral NPs to be stable also under vacuum conditions [see e.g., (Fronzi et al., 2009)].

SCC-DFTB calculations were performed to examine the stabilities of the NP pairs with respect to variation of the angle of rotation of “the second” NP with respect to “the first,” where the axis of rotation is the direction joining the two centers-of-mass of the constituent NPs. Shifting one NP with respect to the other was also considered, leading to an interface which is a continuation of the bulk fluorite structure, i.e., a *coherent* interface. In this way, {111}/{111} and {100}/{100} interfaces were formed. After the SCC-DFTB pre-screening, four final structures emerged from our search for stable (NP)_2_ agglomerates: one with the two NPs joined by {111} faces, and three which were joined by {100} faces; they were subjected to subsequent DFT optimizations. All figures and tables in the following are based on DFT results, except the very last figure which will show results from ReaxFF MD simulations.

#### The {111}/{111} Interface

**Figure 3:**
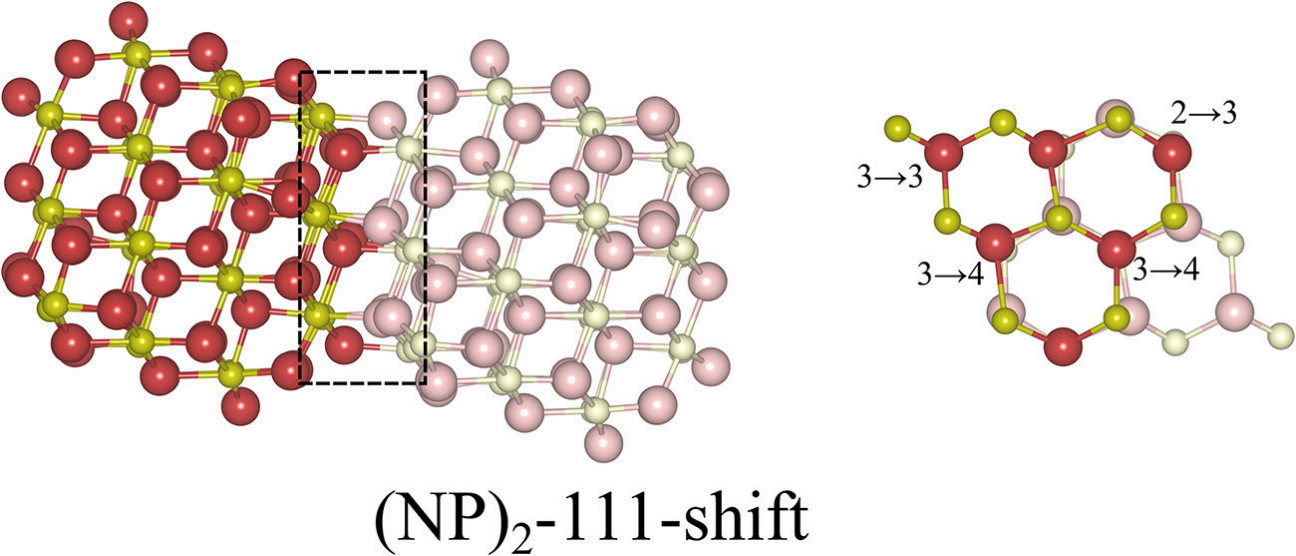
Atomic configuration of the most stable structure that we found for the {111}/{111} interface. It was obtained by shifting one NP with respect to the other. In order to distinguish two NPs clearly, solid and pale colors are used for each NP. The left panel is a side-view, the right panel a cross-sectional view of the interface region, which has been framed in the left figure. The numbers in the right panel indicate the change of coordination number of oxygen ions from the isolated NP to the (NP)_2_ structure. The oxygen ions which are not marked are symmetry-related to one of the marked ones.

Here we note that shifting one NP with respect to the other NP, parallel to the interface, is essential in order to avoid the Coulomb repulsion between ions of the same type; see the cross-sectional view to the right in Figure 3. The most stable structure of the {111}/{111} interface is obtained by shifting one NP to match the stacking sequence of the other NP. As can be seen in Figure 3, the interface structure formed by attaching {111} to {111} facets is coherent. Hereafter, we use the label “(NP)_2_-111-shift” to refer to agglomerated NPs with the {111}/{111} interface structures obtained by shifting.

The interface formation energy, *E*_interface_, is calculated by the following equation:

(1) $$\begin{array}{r} E_{\text{interface}}=E_{\text{tot}}\left[{\left(\text{NP}\right)}_{2}\right]-2\times E_{\text{tot}}\left[\text{NP}\right] \end{array}$$

where *E*_tot_[(NP)_2_] is the total energy of agglomerated NPs with an interface and *E*_tot_[NP] is that of the isolated NP. To aid in the comparison of interface stabilities, especially for interfaces with oxygen vacancies, we will primarily use *E*_interface_ values but also discuss the role of the interface area with normalized interface energies. Our calculated *E*_interface_ of the (NP)_2_-111-shift interface is −8.12 eV, as listed in Table 1. Note that a hexagonal close packed type {111}/{111} interface, which can be considered as a stacking fault, was also tested and found to be far less stable (by 2.72 eV) than the (NP)_2_-111-shift interface.

**Table 1:** Results for the stoichiometric systems, namely for the four stable DFT-optimized NP pairs found in this work, based on the truncated octahedral Ce_40_O_80_ NP.

**Surface/interface label**	**Interfaces involved**	***E*_**interface**_ (eV)**	**Number^a^ of**			**Δ(bonds)^b^**	**Figure**
			**O_**CN = 2**_**	**O_**CN = 3**_**	**O_**CN = 4**_**		
NP(111)	–	–	2	4	0	–	Figure 1
NP(100)	–	–	4	0	0	–	Figure 1
(NP)_2_-111-shift	NP(111)/NP(-1-1-1)	−8.12	0	6	6	+10	Figure 3
(NP)_2_-100-rot15	NP(100)/NP(-100)	−7.19	4	4	0	+4	Figure 4A
(NP)_2_-100-shift	NP(100)/NP(-100)	−6.82	2	4	2	+8	Figure 4B
(NP)_2_-100-rot45	NP(100)/NP(-100)	−5.13	0	8	0	+8	Figure 4C

**Figure 4:**
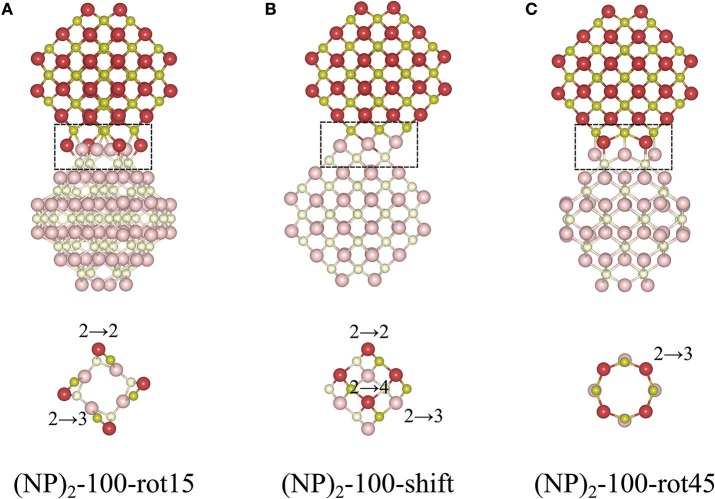
Atomic configurations of the most stable structures of agglomerated Ce_40_O_80_ NPs with an {100}/{100} interface. **(A)** 15° relative rotation of the NPs, **(B)** a relative shift of the NPs, and **(C)** 45° relative rotation of the NPs. In each case, the upper row shows a side-view of the particle, and the bottom row gives a cross-sectional view through the interface region, which has been marked in the top row. The color scheme and the number scheme in the bottom row are the same as in Figure 3.

*Data for the single NP itself is given as references. The table lists the notation, the interfacial energy, E_interface_, calculated according to Equation (1), the number of surface/interface oxygen ions with certain coordination numbers (CNs), the number of new Ce–O bonds created on interface formation, and the figures where the DFT-optimized structures are shown. The interface notation NP(111)/NP(-1-1-1) refers to all symmetry-equivalent combinations, which is of course also true for the (111) surface notation*.

a *Number of surface or interface O ions with CN = 2, CN = 3, CN = 4 (Thus O_CN = 2_, for example, means that all these O atoms in the interface region have exactly two nearest-neighbor Ce ions each)*.

b *Sum of the coordination numbers of all O ions in the interface region (they are depicted in Figures 3, 4) minus the sum of the coordination numbers of the same atoms in the isolated NPs. Neighbors within a distance limit of 2.6 Å were counted*.

#### The {100}/{100} Interface

The protocol for finding stable {100}/{100} interfaces included a scan of the angle of rotation of one NP with respect to the other from 0 to 45° with an increment of 1°. Following this pre-screening, three distinctly different stable structures were found, c.f. Figure 4 and Table 1. They are either rotated by 15 or 45° with respect to each other, or by shifting one NP in a direction perpendicular to the interface normal by half of the lattice parameter so as to match the bulk fluorite structure. We will refer to them as “(NP)_2_-100-rot15,” “(NP)_2_-100-rot45,” and “(NP)_2_-100-shift” in the following. Thus, our results show that the {100}/{100} interface structures can be either coherent or incoherent: the {100}/{100} interface obtained by shifting is coherent while the {100}/{100} interfaces obtained by rotating by 15 or 45° are incoherent. In the literature, it has been suggested (Sk et al., 2014) that the formation of {100}/{100} interfaces requires that one side of a truncated ceria surface be cerium-terminated (having oxygen vacancies at a {100} surface) while the opposite side needs to be oxygen-terminated in order to match the crystal ordering when two particles agglomerate. However, this is a restriction following the use of periodic boundary conditions, where one NP formed an interface between a cerium-terminated surface and an oxygen-terminated surface of the same NP. When we give full freedom to the relaxation and formation of the {100}/{100} interface, such constraints do not exist.

The calculated *E*_interface_ values for the agglomerated NPs with the 15 and 45° rotation is −7.19 and −5.13 eV, respectively, and for “(NP)_2_-100-shift”, it is −6.82 eV. Thus, if the NPs are stoichiometric, the {111}/{111} interface (−8.12 eV) is more stable than the {100}/{100} interface structures, likely due to the denser and larger contact areas of the former. It is important to note that for the reported interface energies, the contact areas are very different. The approximate area of the (111) facet on the free Ce_40_O_80_ NP is 43.8 Å^2^ and that of the (100) facet is only 13.3 Å^2^. This implies that the normalized interface energy per interface area will be much larger in case of the {100}/{100} interface. For the most stable {111}/{111} and {100}/{100} interfaces on the (NP)_2_ agglomerate, we get −2.97 J/m^2^ (−0.19 eV/Å^2^) and −8.66 J/m^2^ (−0.54 eV/Å^2^), respectively. It is interesting to compare these values with the calculated surface energies of 0.71 J/m^2^ and 1.54 J/m^2^ for the extended (111) and (100) surfaces, respectively (Kullgren et al., 2017). The calculated surface energy of the (100) surface in Kullgren et al. (2017) was obtained for a reconstructed (100) surface where half of the terminal O ions on the (100) surface were moved to the opposite face to compensate the polarity of the surface. The normalized interface energy in the limit of an infinite interface will be twice the negative surface energy, and thus becomes −1.42 and −3.08 J/m^2^, respectively. We note that the calculated normalized nano-interface energies reveal stronger adhesion by at least a factor of 2 compared to the limiting values. This reflects the fact that the surfaces on the small Ce_40_O_80_ NP mainly consist of rim atoms. Thus, even though the {111}/{111} interface on a total energy scale is more stable than the {100}/{100} interfaces, the latter has a stronger adhesion per unit area, and it may very well form in experiments through an initial first approach via the OA mechanism, and then become kinetically trapped (Si et al., 2006; Tan et al., 2011; Florea et al., 2013).

**Figure 5:**
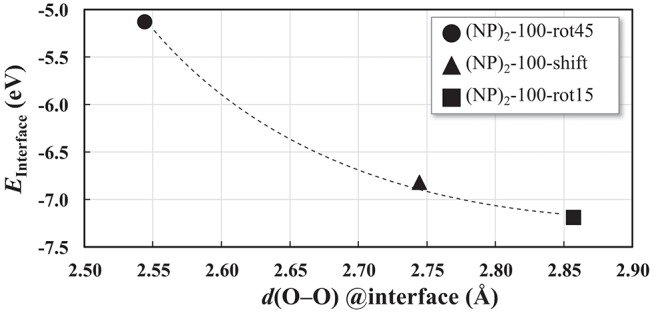
*E*_interface_ as a function of the O–O nearest-neighbor distances averaged over the 8 O ions in the interface region of each of the three “100” interfaces displayed in Figure 4. The curve is a guide to the reader's eye.

The interatomic distance within each nearest neighbor (NN) pair, Ce—O, Ce—Ce, and O—O at the rim of the interface of the three {100} systems, (NP)_2_-100-rot15, -rot45, and -shift, are further analyzed here. First, let us discuss the NN distances of all constituent pairs at the {100} facets that are *not* involved in the interface formation. They are found to be identical with those of the isolated single Ce_40_O_80_ NP; this indicates that the effect of interface formation on the structural distortion is localized at the interface. Intuitively, one could imagine that the interface stability will correlate with the number of bonds formed upon agglomeration. We have therefore calculated coordination numbers and coordination number differences, Δ(bonds), for the all studied interfaces, c.f. Table 1. We note that while this hypothesis can be used to explain why (NP)_2_-100-rot15 is the most stable interface among the three {100} systems, it fails to discriminate between the different {100}/{100} interfaces. The calculated Δ(bonds) for (NP)_2_-100-rot15, -rot45, and -shift is +4, +8, and +8, respectively. Here, the NN distances of Ce—Ce and O—O, which will cause repulsive forces and thereby result in a less negative *E*_interface_ (less stable interface), give a better correlation, c.f. Figure 5, which shows the calculated *E*_interface_ vs. average O—O NN distance. The average O—O NN distance at the {100} facet in the isolated Ce_40_O_80_ NP is 2.56 Å, and at the interfaces the resulting distances are 2.86, 2.54, and 2.74 Å for (NP)_2_-100-rot15, -rot45, and -shift, respectively. For (NP)_2_-100-rot45 which is the least stable interface structure among our model interfaces, all the distances of O—O NN pairs at the interface are shorter than 2.56 Å. On the other hand, the interface formation by rotating 15° eliminates these short O—O NN pairs at the interface. We conclude that the larger stability of (NP)_2_-100-rot15 is attributed to the stretching of the short O—O pairs at the interface, which mitigates repulsion.

**Figure 6:**
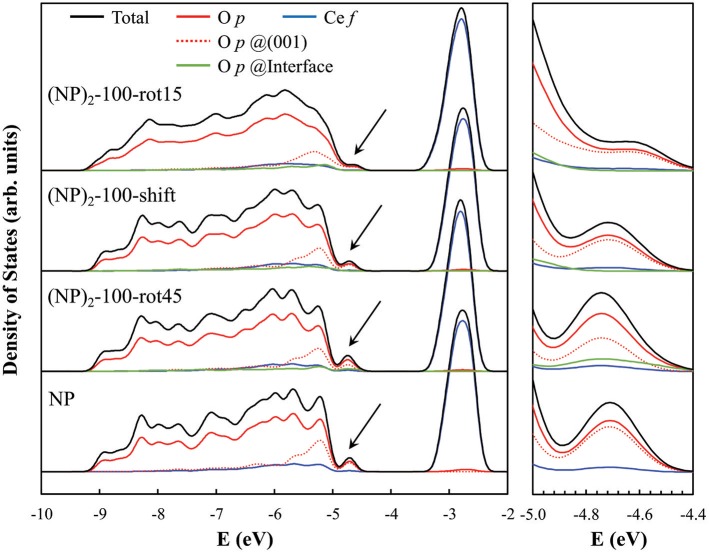
Electronic PDOS for the three “100”-connected (NP)_2_ systems as well as for the single NP (as a reference). The PDOS diagrams were aligned with respect to the core levels of the O atoms at the center of each NP system. The black arrows in the left panel indicate the surface states induced by the terminal O^2−^ ions at the free {100} facets of the single NP and (NP)_2_ systems, and especially so at the (NP)_2_-100-rot45 interface (see text); these states are magnified in the rightmost panel. The computed band gaps [VB(O 2*p*) → CB(Ce 4*f*)] for (NP)_2_-100-rot15, -shift, -rot45 and the isolated NP are 1.25, 1.49, 1.36, and 1.53 eV, respectively.

The presence of O—O pairs with short distances affects the electronic properties of agglomerated ceria NPs. This is seen in the electronic projected density of states (PDOS) for the three {100}/{100} interface systems when compared to the isolated NP (see Figure 6). Here, most notably, we find a clear peak at the valence band maximum in the PDOS for the isolated NP and (NP)_2_-100-rot45 interface. Interestingly, the magnitude of the peak is found to be smaller in case of the (NP)_2_-100-shift and -rot15 interfaces (see the black arrows in Figure 6). From the PDOS, it is clearly seen that this peak primarily originates from terminal O ions at the (100) facets (see the dashed red lines in Figure 6). We find that they are preserved for (NP)_2_-100-rot45 whereas in the (NP)_2_-100-shift and -rot15 interfaces, they are effectively quenched (compare solid green lines in Figure 6). Thus, in a sense, the (NP)_2_-100-rot45 interface structure may be regarded as *electronically* analogous to the free {100} facets of isolated Ce_40_O_80_ NPs. For (NP)_2_-100-rot15 and -shift, on the other hand, the surface states are significantly reduced by the interface formation.

### Oxygen Chemistry

Oxygen vacancies play a central role in the redox chemistry of ceria and we have investigated whether and how the oxygen vacancy formation energy, *E*_vac_, is affected by the creation of an interface between the NPs. *E*_vac_ is here calculated in the usual way, i.e., as

(2) $$\begin{array}{r} E_{\text{vac}}=E_{\text{tot}}\left[\text{NP}+n{\text{V}}_{\text{O}}\right]-E_{\text{tot}}\left[\text{NP}\right]+n\times \frac{1}{2}E\left[{\text{O}}_{2}\left(g\right)\right] \end{array}$$

where *E*_tot_[NP + *n*V_O_] and *E*_tot_[NP] are the total energies of the system with and without the number of oxygen vacancies, *n*V_O_, respectively. *E*[O_2_(*g*)] is the energy of an O_2_ molecule in its ground triplet electronic state.

First we give a general remark, namely that we find that in all cases, for the interfaces as well as for the single NP, the oxidation states of the apex Ce ions (determined from the 4*f*-electron occupancy) change from 4+ to 3+ after the oxygen vacancy formation. The apex Ce ions are marked in Figure 1 for the isolated NP and are present in all the (NP)_2_ systems as well but are not so easy to discern in the figures. The locations of the excess electrons created at the interface is consistent with several previous single-NP studies in the literature, which reported that the two Ce^3+^ ions are preferentially located at the apex sites where the coordination numbers of the Ce ions are the lowest (see Loschen et al., 2007, 2008; Kullgren et al., 2013; Sk et al., 2014).

**Figure 7:**
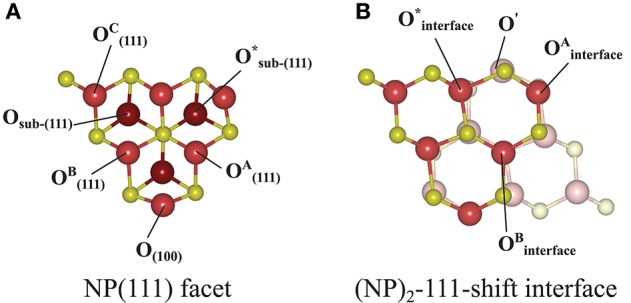
Atom labeling scheme used in the text and in Table 2 for discussion of oxygen vacancy formation at **(A)** the {111} facet of the isolated Ce_40_O_80_ NP and at **(B)** the (NP)_2_-111-shift interface formed from it. The same color scheme as in Figure 3 has been used, except that the sub-surface oxygens in **(A)** are colored dark red. In both **(A)** and **(B)**, the left-most Ce atom in the upper row and the right-most Ce atom in the bottom row in **(B)** are the “apex Ce” marked in Figure 1.

Let us now consider oxygen vacancy formation at different surfaces and sub-surface positions on an *isolated NP* as shown in Figure 1. The vacancy will be labeled according to the facet involved and the label of the surface of sub-surface O removed, as listed in the second column in Table 2. The resulting *E*_vac_ values are also given in the table. The smallest *E*_vac_ value, i.e., the energetically most stable site for an oxygen vacancy to be formed in an isolated NP is the 2-coordinated O^2−^ site at the {100} facet (“O_(100)_” in Figure 7A) with *E*_vac_ = +0.55 eV, which is in good agreement with the previous DFT work published by the group of Neyman et al., showing that a low-coordinated oxygen vacancy formation costs the least energy (Migani et al., 2010). Oxygen vacancies at the {111} facet (see Figure 7A) fall in the range from +1.50 to +1.91 eV depending on their vicinity to the {100} facets; the further from the {100} facet, the higher the *E*_vac_ value.

**Table 2:** Vacancy formation energies, *E*_vac_, for the formation of one and two vacancies at the surface/interface according to Equation (2).

**System**	**Label of 1st removed O**	**Coordination number**	***E*_vac_, 1st (eV)**	***E*_vac_, 2nd (eV)**
NP(111)	(111) (A)^a^	3	+1.50	Not done
	(111) (B)^a^	3	+1.75	Not done
	(111) (C)^a^	3	+1.91	Not done
	(100)^a^	2	+0.55	Not done
	Sub-(111)^a^	4	+1.83	Not done
(NP)_2_-111-shift	Interface (A)^b^	3	+0.59	+0.72
	Interface (B)^b^	4	+1.31	Not done
(NP)_2_-100-rot15	Interface (A)^c^	2	+0.28	+0.26
	Interface (B)^c^	3	+0.69	Not done
(NP)_2_-100-shift	Interface (A)^d^	4	+0.24	+0.56
	Interface (B)^d^	2	+0.31	Not done
	Interface (C)^d^	3	+0.37	Not done
(NP)_2_-100-rot45	Interface^e^	3	−1.53	+0.11

*For the (NP)_2_ systems, a 2nd vacancy was created by removal of each of the remaining interface O ions in turn, but only one of them at a time; the lowest value found is reported as the 2nd E_vac_ in the table*.

a *Figure 7A*.

b *Figure 7B*.

c *Figure 8A*.

d *Figure 8B*.

e *Figure 8C*.

We find that the most preferable site for an oxygen vacancy to form at *the {111}/{111} interface* is at a 3-coordinated O^2−^ site (labeled ${\text{O}}_{\text{interface}}^{\text{A}}$ in Figure 7B) with a formation energy of +0.59 eV which is similar to the corresponding site in the isolated NP. For ${\text{O}}_{\text{interface}}^{\text{B}}$, *E*_vac_ is +1.31 eV. When an oxygen vacancy was formed at ${\text{O}}_{\text{interface}}^{*}$, the vacancy was found to be healed by O′ which was originally located behind the adjacent Ce, which results in the same structure as when the vacancy was created at ${\text{O}}_{\text{interface}}^{\text{A}}$. We conclude that for a pair of truncated octahedral NPs joined by an {111}/{111} interface, which is coherent, the interface formation has none (or a very small) effect on the oxygen vacancy formation capability at the interface.

**Figure 8:**
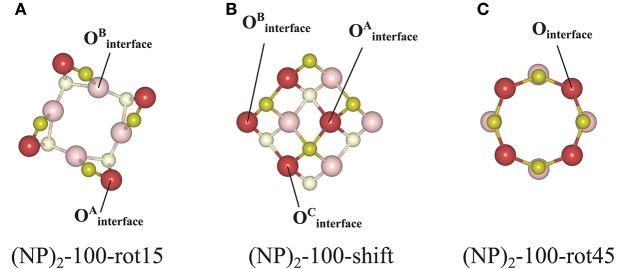
Atom labeling scheme used in the text and in Table 2 for discussion of oxygen vacancy formation at **(A)** the (NP)_2_-100-rot15, **(B)** the (NP)_2_-100-shift, and **(C)** the (NP)_2_-100-rot45 interface structures.

For *the {100}/{100} interfaces*, the structures and oxygen site labeling schemes are shown in Figure 8 and their coordination numbers and *E*_vac_ values are given in Table 2. We find that here the oxygen vacancy formation behavior is different from both that of the isolated NP and that of the {111}/{111} interface. For the most stable configuration among the {100}/{100} interface structures, (NP)_2_-100-rot15, the lowest *E*_vac_ value (+0.28 eV) is about half of the lowest value for the isolated NP, which was already small (+0.55 eV) (c.f. Figure 8A). In fact, all the {100}/{100} interfaces display very small *E*_vac_ values despite the fact that the interfaces themselves are quite stable. The reason is no doubt connected to the fact that new bonds are formed between the NPs but the NN coordination numbers are modest in these structures: enough to hold the particles strongly together but not enough to hang on to O ions if the opportunity for reduction arises. This is true not only at the rims of these interfaces, but throughout the cross-section, as the coordination number is maintained also in the middle of the cross-section.

The ultimate oxygen lability is demonstrated by the (NP)_2_-100-rot45 interface which has a large negative *E*_vac_, −1.53 eV, meaning that, thermodynamically speaking, this oxygen vacancy would be formed spontaneously at the interface (and have a stabilizing effect on the interface). A second oxygen can also easily be removed, at hardly any cost (c.f. Table 2). The *E*_vac_ values of the second oxygen vacancy lies in the range from +0.11 to +0.56 eV for all the {100}/{100} interface structures. Under alternating reducing and oxidizing conditions, i.e., when the chemical potential of oxygen changes, *E*_vac_ will change accordingly. At some point, *E*_vac_ will turn negative and vacancies will stabilize the interface. Thus, reducing conditions may promote the stability of the interface significantly by oxygen removal from the interface.

The decreased *E*_vac_ value at {100}/{100} interfaces can be understood by examining the average NN O—O distance at the interface. We find that the occurrence of short O—O pairs at the interface (discussed above) diminishes after the oxygen vacancy formation. For example, the average NN O—O distance of (NP)_2_-100-rot45 increases significantly, from 2.54 to 2.85 Å, when an oxygen vacancy is formed at the interface. It is then also observed that the two Ce^3+^ ions are generated at the apexes of the agglomerated NPs. We have found (not shown here) that these generated Ce^3+^ ions can be further utilized as adsorption sites for incoming oxygen molecules from the surroundings, similar to the phenomenon of *supercharged oxygen storage capacity* discussed earlier in the literature for ceria NPs (Kullgren et al., 2013; Renuka et al., 2015).

**Figure 9:**
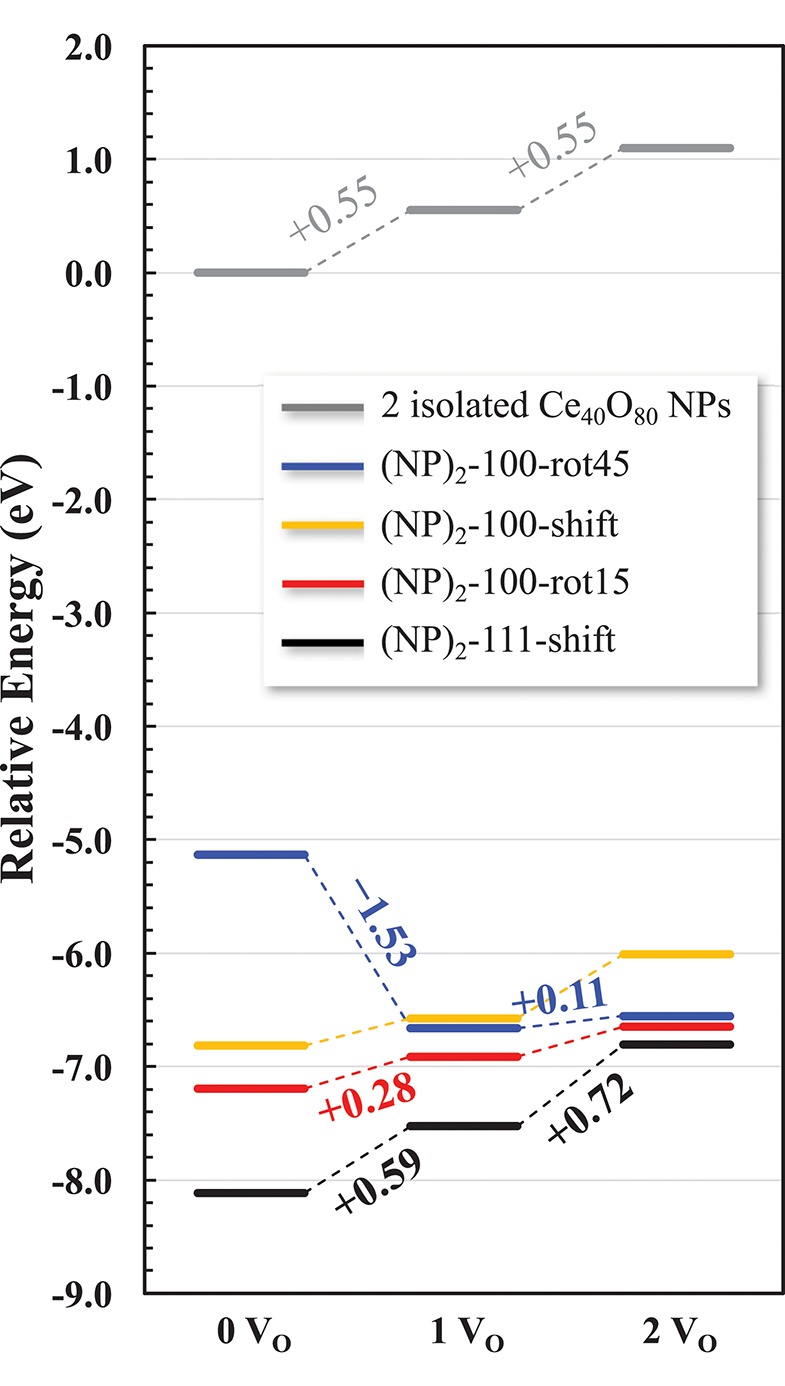
Relative energy diagram for oxygen reduction of agglomerated NPs. Numbers along the pathway denote the corresponding *E*_vac_. Two isolated NPs constitute the reference system.

In summary, we find that {100}/{100} interface formation can lower *E*_vac_ significantly. In other words, the interface formation eliminates the areas of reactive (100) surfaces but promotes a more facile oxygen extraction, which in turn may promote an increased low-temperature oxygen storage capacity of ceria via the so called supercharging mechanism (Kullgren et al., 2013). Figure 9 summarizes the relative energies of the interface structures considered in this work as a function of increasing number of oxygen vacancies. The reference system is two isolated NPs at infinite distance, and their oxygen vacancies are created by removing neutral O atoms from a {100} facet of each NP. While the {111}/{111} interface is more stable for the stoichiometric cases, we find that the {100}/{100} interface promotes a lower *E*_vac_ compared to the {111}/{111} interface. Interestingly, in the same comparison, oxygen vacancy formation at the {111}/{111} interface slightly increases *E*_vac_. As a result, in the presence of two oxygen vacancies, the stabilities of {100}/{100} interface structures, here (NP)_2_-100-rot15 and -rot45, become comparable with that of the {111}/{111} interface (see Figure 9). This result paves the way not only for understanding the engineering of the interface structure of nanoceria for improved low-temperature redox activity, but also for suggesting experimental guidelines to control a growth direction of ceria nanowires. For example, our results suggest that under oxidizing conditions, a preferential growth direction of agglomerated NPs would be 〈111〉 while the {100}/{100} interface can be formed dominantly under reducing conditions.

### Extrapolation to Larger Systems

**Figure 10:**
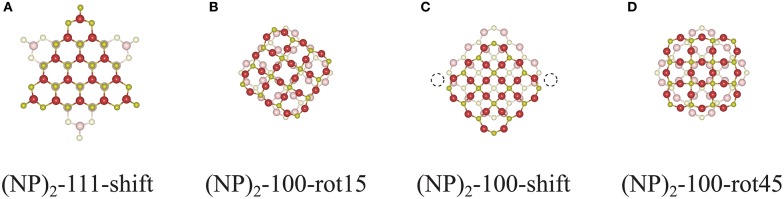
Cross-sectional views of the interface structures of **(A)** (NP)_2_-111-shift, **(B)** (NP)_2_-100-rot15, **(C)** -shift, and **(D)** -rot45 of two unrelaxed agglomerated Ce_132_O_264_ NPs (taken from truncated DFT bulk structures).

One might raise a question whether the interface construction procedure used in this work will also apply to larger systems and what role the interface area plays. To answer these questions, we constructed interfaces between two Ce_132_O_264_ NPs as shown in Figure 10. The {111}/{111} interface can still be formed following a continuation of bulk fluorite (see Figure 10A), and one might expect that the interface formation will be similar to that of the smaller system, Ce_40_O_80_, albeit with a larger area of overlap. However, for {100}/{100} interfaces, the interface construction by rotation does not work since it requires a large rearrangement of constituent ions at the interface which will induce a distortion of the structure. For example, since the interface structure of (Ce_132_O_264_)_2_-100-rot15 (Figure 10B) contains overlapping Ce and O ions at the center of the interface, it is impossible to avoid the repulsion between these unless the whole interface structure is distorted, which obviously requires a huge amount of energy. “Overlapping ions” here means that if the interface region is seen in a projection perpendicular to the interface, then the Ce ions in the two constituent NPs would (almost) overlap with each other, and similarly for the O ions. Interface construction by shifting also suffers from O ion overlaps as the interface area becomes larger. For a (Ce_132_O_264_)_2_-100-shift NP dimer, constructed as shown in Figure 10C, there are 36 overlapping O ions at the interface, but only two sites are available for Ce—O bonding (dashed circle in Figure 10C). To prevent this overlapping and produce a coherent bulk fluorite structure at the interface as was achieved for (Ce_40_O_80_)_2_-100-shift, at least 16 O ions need to be removed at the interface. Needless to say, the stoichiometric interface structure of (Ce_132_O_264_)_2_-100-rot45 as shown in Figure 10D would be very unstable due to the overlapped ions at the interface. However, they could be stabilized at reducing conditions by oxygen removal from the interface.

For the large NPs as well as for the smaller ones, we have also performed ReaxFF molecular dynamics (MD) simulations addressing the dynamics of the agglomeration process. The parameter set of ReaxFF used here was derived by us from DFT data (Broqvist et al., 2015). Further technical details about the validation of the parameters of ReaxFF are presented in the Supplemental Material. Two simulations extending over 5 ns at 400 K using the NVT ensemble, one with 10 Ce_40_O_80_ NPs and one with 10 larger Ce_132_O_264_ NPs, were performed in the current study. The former system consists of particles exposing four small {100} facets each (c.f. Figure 1), while the latter represents a system of particles exposing only one, albeit larger, {100} facet per particle. One might say the MD simulations are too short to capture the entire agglomeration process. However, they are long enough to include the coalescence and initial restructuring of the interfaces. In the case of a low particle density (as we have here) we expect that the effective barrier to transform a meta-stable {100}/{100} interface into the energetically more stable {111}/{111} interface must be bound by the interface energy of the former. The magnitude of the interface energy is larger than 5 eV which is large enough to permit long-lived interfaces, even if they happen to be meta-stable ones. The real barrier could obviously be quite different since particles forming the interface may slide or rotate while still maintaining contact. One aim with these MD simulations is therefore to look for indications of dynamical changes in the interface structures formed.

**Figure 11:**
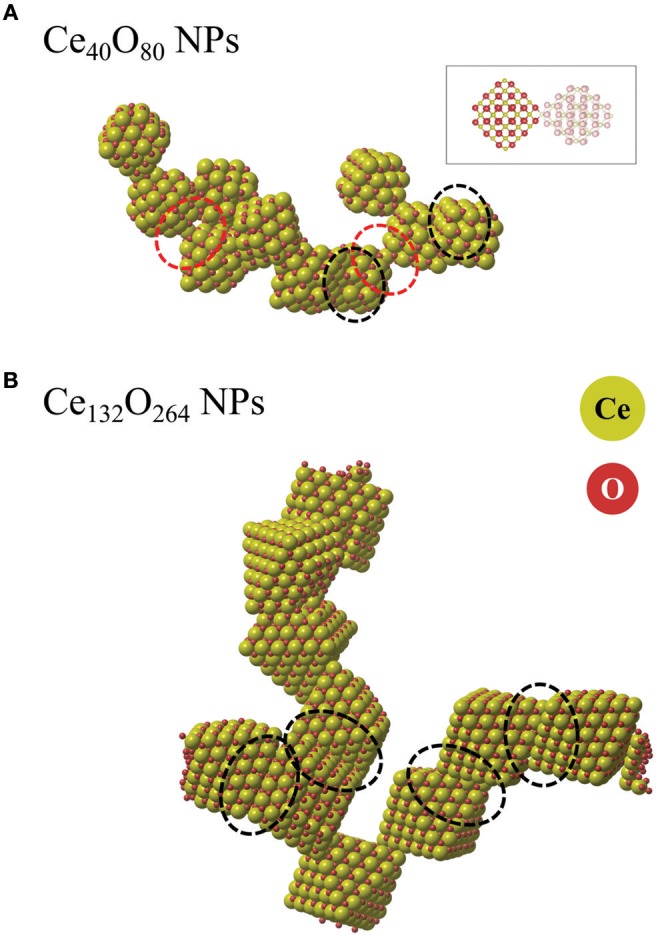
Snapshots from ReaxFF MD simulations of agglomerated NPs of **(A)** Ce_40_O_80_ and **(B)** Ce_132_O_264_. The ionic radius of Ce ions was enlarged for the sake of clarity. Red and black dashed ovals indicate apex/{100} interfaces and {111}/{111} interfaces, respectively. The inset indicates the atomic configuration of an apex/{100} interface.

Figures 11A,B compare representative snapshots from the two simulations. We first note that the Ce_40_O_80_ NPs form an isolated agglomerate while the Ce_132_O_264_ NPs form an extended two-dimensional web. In both cases, we observe that various kinds of interfaces are formed, not only the energetically stable {111}/{111} interfaces as we described in the earlier section, but also {111}/{100}, apex/{100}, and partially matched {111}/{111} interfaces. This observation is in line with the theoretical findings of Fichthorn and co-workers (Alimohammadi and Fichthorn, 2009; Raju et al., 2014) who showed that titania NPs prefer to aggregate in the direction of approach in vacuum, which suggests that the interface formation may not always follow the thermodynamically most stable pathway. For example, in the system of the smaller particles (Ce_40_O_80_), we do not observe any formation of the energetically stable {100}/{100} interfaces, but instead find the coherent {111}/{111} interfaces, incoherent {111}/{100} interfaces, and apex/{100}. We attribute this fact to the strong variation in interface energy with respect to particle rotation, which implies that particles have to meet at “just the right” angle to form the {100}/{100} interface. We observe two interfaces where the apex ion of one particle binds to the center of a {100} facet of another particle (apex/{100}). We note the similarity of this structure to the interface presented in the theoretical work by Sk et al. (2014), although, in our case the two particles are rotated with respect to each other. In this structure (Figure 11A), a positively charged Ce ion at one apex is bonded to a square of negatively charged O ions on the {100} facet. The interface is seen to readily rotate during the MD simulation. For comparison, we calculated *E*_interface_ of the apex/{100} interface energy using Equation (1) at the DFT level to be −1.87 eV. This value is significantly smaller in magnitude than that of the {100}/{100} interfaces which range from −5.1 to −7.2 eV. Nevertheless, the energy is not small enough to rule out that apex/{100} interfaces, once formed, could have a substantial life time, clearly longer than the short simulation time used here. The snapshot from the MD simulation of the Ce_132_O_264_ system shown in Figure 11B dominantly displays the formation of {111}/{111} interfaces. This is in good agreement with an experimental finding (c.f. Figure 7B in Florea et al., 2013). As discussed for overlapping ions of larger NPs above, the fact that {100}/{100} interfaces are not found in our agglomerate of Ce_132_O_264_ NPs is likely due to the repulsion between overlapping Ce and O ions, respectively. However, under reducing conditions such interfaces might be more likely to form, which is an interesting topic for further study.

## Conclusions

In this work, we addressed the effects that agglomeration may have on the stability and redox activity of ceria NPs. We applied a computational protocol based on a multiscale simulation approach combining three levels of theory, namely DFT, SCC-DFTB and ReaxFF. In particular, we have studied the formation of Ce_40_O_80_ pairs using DFT and SCC-DFTB, and larger agglomerates of Ce_40_O_80_ or Ce_132_O_264_ NPs in MD simulations with ReaxFF.

The most stable {111}/{111} interface structure was found to be coherent, i.e., it exhibits a continuation of the bulk structure, whereas the stable {100}/{100} structures can be either coherent or incoherent. A systematic study of the implications of interface formation on the oxygen chemistry revealed that (i) {111}/{111} interface formation has only a very small effect on the redox activity, and (ii) *E*_vac_ decreases significantly for oxygen atoms in the vicinity of {100}/{100} interfaces. We conclude that while interface formation eliminates reactive {100} facets, it promotes an enhanced low-temperature redox activity involving the extraction of lattice oxygen; the net result might be an overall increased oxygen storage capacity of ceria.

## Author Contributions

B-HK has performed the simulations and drafted the first version of the manuscript. All other authors have been equally involved in the planning of the work and in the finalization of the text.

### Conflict of Interest Statement

The authors declare that the research was conducted in the absence of any commercial or financial relationships that could be construed as a potential conflict of interest.
