# Supplementary material for: Multiscale Modeling of Agglomerated Ceria Nanoparticles: Interface Stability and Oxygen Vacancy Formation
Source: Front Chem. 2019 May 22;7:203. doi: 10.3389/fchem.2019.00203 (PMC6538807; doi:10.3389/fchem.2019.00203)
Supplement: Supplementary file 1 [file Data_Sheet_1.pdf]

Supplemental material of: Multiscale modelling of agglomerated ceria nanoparticles: interface stability and oxygen vacancy formation

Byung-Hyun Kim[1,2], Jolla Kullgren[1*], Mattthew Wolf[1], Kersti Hermansson[1*], and Peter Broqvist[1*]

1 Department of Chemistry–Ångström Laboratory, Uppsala University, Box 538, S-751 21, Uppsala, Sweden

2 Platform Technology Laboratory, Korea Institute of Energy Research, Daejeon, Republic of Korea

* *jolla.kullgren@kemi.uu.se, kersti@kemi.uu.se, peter.broqvist@kemi.uu.se*

Table S-I. The calculated energies such as oxygen vacancy formation, and interface formation using SCC-DFTB and DFT methods

**Structure**		**SCC-DFTB (eV)**	**DFT (eV)**	**Error (eV)**
NP	E_{vac}, 1st @(100)	+0.47	+0.55	–0.08
	E_{vac}, 2nd @(100)	+1.77	+1.53	+0.24
	E_{vac}, 1st @(111) (A)	+1.18	+1.50	–0.32
	E_{vac}, 1st @(111) (B)	+1.40	+1.75	–0.35
	E_{vac}, 1st @(111) (C)	+1.67	+1.91	–0.24
	E_{vac}, 1st @sub-(111)	+2.28	+1.83	–0.45
$(NP)_2$-111-shift	$E_{Interface}$	–9.50	–8.12	–1.38
	E_{vac}, 1st @Interface (A)	+0.74	+0.59	+0.15
	E_{vac}, 2nd @Interface (A)	+0.55	+0.72	+0.17
	E_{vac}, 1st @Interface (B)	+0.98	+1.31	–0.33
$(NP)_2$-100-rot15	$E_{Interface}$	–8.85	–7.19	–1.66
	E_{vac}, 1st @Interface (A)	+0.04	+0.28	–0.24
	E_{vac}, 2nd @Interface (A)	+0.20	+0.26	–0.06
	E_{vac}, 1st @Interface (B)	+0.03	+0.69	–0.63
$(NP)_2$-100-rot45	$E_{Interface}$	–6.38	–5.13	–1.25
	E_{vac}, 1st @Interface	–2.32	–1.53	–0.79
	E_{vac}, 2nd @Interface	+0.15	+0.11	+0.04
$(NP)_2$-100-shift	$E_{Interface}$	–8.00	–6.82	–1.18
	E_{vac}, 1st @Interface (A)	–0.29	+0.24	–0.53
	E_{vac}, 2nd @Interface (A)	+1.75	+0.56	+1.19

Reactive Force Field (ReaxFF)

The parameters for Ce–O system proposed by P. Broqvist *et al.* [1] was rigorously tested. We found that two local minima exist along the diffusion path of an oxygen vacancy, which results in a problematic description of oxygen vacancy diffusion in bulk ceria. Thus, the parameters were modified by simply turning off the pi bonding of Ce–O which was identified as the main reason for overestimating the short bond. **Table S-II** summarizes the benchmark results which are in a good agreement with the previous calculations. All MD calculations were performed by LAMMPS code [2].

Table S-II. Optimized lattice parameter, bulk modulus, oxygen vacancy formation energy, and surface energies for low index surfaces in the Fluorite crystal phase.

		**Original**	**This work**	**Reference (PBE+U)**
Lattice Parameter (Å)		5.49	5.50	5.49
Bulk Modulus (GPa)		192	205	179
$D_e(O_2)$ (eV)		5.46	5.46	5.7
E_{vac} (eV)		2.91	3.04	3.3
Surface Energy (J/m^2)	(111)	0.84	0.86	0.71
	(110)	1.06	1.11	1.09
	r-(001)	1.62	2.41	1.54
	(001)	2.5	3.41	-

[1] P. Broqvist, J. Kullgren, M. J. Wolf, A. C. T. van Duin, and K. Hermansson, J. Phys. Chem. C 119, 13598 (2015).

[2] S. Plimpton, J. Comp. Phys. 117, 1 (1995).
